# Sustainable industrial crop-based strategies for cocoa butter substitutes: advances in plant, microbial, and lipid engineering

**DOI:** 10.1016/j.fochx.2026.104187

**Published:** 2026-07-11

**Authors:** Yuxin Zou, Yan Lu, Changquan Zhang, Baolong Zhang, Qing Liu

**Affiliations:** a School of Food and Biological Engineering, Jiangsu University, Zhenjiang, Jiangsu Province 212013, China; b Jiangsu Co-Innovation Centre for Modern Production Technology of Grain Crops, Yangzhou University, Yangzhou 225009, China; c Zhongshan Biological Breeding Laboratory, Jiangsu Academy of Agricultural Sciences, Nanjing, Jiangsu Province 210014, China

**Keywords:** Cocoa butter substitutes, Triacylglycerols, Enzymatic interesterification, Microbial oils, Plant biotechnology

## Abstract

Cocoa butter (CB) is the defining lipid of chocolate, valued for its unique triacylglycerol (TAG) composition and polymorphic behaviour that confer gloss, snap, and rapid melt-in-mouth properties. Increasing volatility in cocoa (*Theobroma cacao*) supply, driven by climate pressures and rising demand, has intensified interest in cocoa butter substitutes (CBS). This review summarizes recent advances across the main technological routes for CBS development. Conventional sources such as palm mid-fractions and shea butter provide compositional compatibility, while enzymatic interesterification and fractionation can partially reshape TAG profiles towards CB-like structures. Engineered oleaginous yeasts have also been developed to produce CBS lipids through metabolic rewiring and heterologous acyltransferase expression, although economic constraints remain. Plant metabolic engineering has generated seed oils enriched in oleic, stearic, or palmitic acids with reduced polyunsaturated fatty acids. However, reproducing the balanced palmitic–stearic–oleic composition and stereospecific TAG architecture of CB in scalable crop systems remains a major challenge.

## Highlights

• Cocoa butter functionality is defined by precise POP–POS–SOS stereochemistry.
• Enzymatic interesterification enables high-fidelity cocoa butter equivalents.
• Microbial cell factories provide programmable SOS-type TAG synthesis.
• Plant metabolic engineering achieves HO/HS/HP traits but faces assembly limits.
• Industrial-scale CBS requires integration of precision, sustainability, and regulation.

## 1 Introduction

**Fig. 1:**
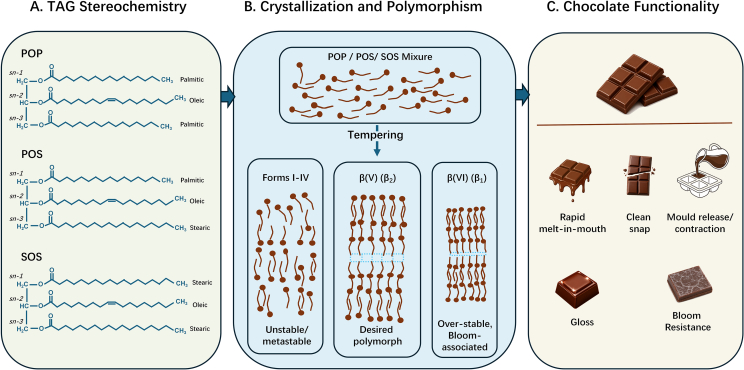
Structural basis of cocoa butter polymorphism and chocolate functionality. (A) Cocoa butter is dominated by the symmetric saturated–unsaturated–saturated TAGs POP, POS, and SOS. In these molecules, oleic acid is positioned at *sn*-2, while palmitic and/or stearic acids occupy the *sn*-1 and *sn*-3 positions. This stereochemical arrangement provides the molecular basis for efficient crystal packing. (B) During tempering, the POP/POS/SOS mixture crystallizes into different polymorphic forms. Unstable or metastable forms I–IV have disordered packing and can transform during storage. Correct tempering favours formation of β(V), or β₂, which has ordered packing and is the desired crystal form in chocolate. The more stable β(VI), or β₁, form may develop during ageing and is commonly associated with fat bloom. (C) Formation of β(V) crystals gives chocolate its key quality attributes, including gloss, clean snap, mould release/contraction, rapid melt-in-mouth, and improved bloom resistance. The chemical structures shown are simplified schematic representations for conceptual illustration only and are not intended to depict exact molecular structures. Abbreviations: TAG, triacylglycerol; POP, 1,3-dipalmitoyl-2-oleoyl-glycerol; POS, 1-palmitoyl-2-oleoyl-3-stearoyl-glycerol; SOS, 1,3-distearoyl-2-oleoyl-glycerol.

Cocoa butter (CB) is the defining lipid of chocolate and confectionery, conferring the characteristic gloss, snap, and rapid melt that underpin product quality and consumer perception. These functional properties arise from its highly ordered triacylglycerol (TAG) composition and polymorphic behaviour, making CB unusually difficult to replicate with alternative fats. As global chocolate consumption expands, demand for CB continues to increase. Global chocolate consumption reached approximately 8.13 million tonnes in 2021, with the market valued at USD 113.6 billion, while cocoa and chocolate trade transactions were estimated at USD 46.61 billion in 2021 and projected to reach USD 67.88 billion by 2029 (Wang & Dong, 2024). At the processing level, global cocoa grindings remained substantial at 4.81 million tonnes in 2023/24, although they declined to an estimated 4.60 million tonnes in 2024/25 under high input-cost pressure (Santos et al., 2024). CB is derived exclusively from the lipid-rich seeds of cocoa (*Theobroma cacao* L.), a perennial tropical crop cultivated within a narrow equatorial belt. Unlike most vegetable oils, CB is dominated by a limited set of symmetric monounsaturated TAGs, principally 1,3-dipalmitoyl-2-oleoyl-glycerol (POP), 1-palmitoyl-2-oleoyl-3-stearoyl-glycerol (POS), and 1,3-distearoyl-2-oleoyl-glycerol (SOS), in which oleic acid (C18:1^Δ9^) is preferentially esterified at the *sn*-2 position and palmitic (C16:0) or stearic acid (C18:0) occupies the *sn*-1/*sn*-3 positions (Table 1; Fig. 1). This highly ordered TAG architecture underpins CB's sharp melting profile, crystallization behaviour, and ability to form the desirable β(V) polymorph, thereby contributing to the gloss, snap, bloom resistance, mould release, and rapid melt-in-mouth properties of chocolate (Ghazani & Marangoni, 2021; Jahurul et al., 2013; Lipp & Anklam, 1998). At the same time, cocoa production is increasingly vulnerable to climate change, disease outbreaks, land-use constraints, and persistent socio-economic challenges in producing regions (Asante et al., 2025; Fountain & Hütz-Adams, 2025). These pressures expose the fragility of a supply chain dependent on a geographically restricted crop with long breeding cycles and limited agronomic flexibility. Ensuring long-term CB availability is therefore not only a matter of food-processing innovation but also an agricultural and industrial systems challenge.

**Table 1:** Major triacylglycerol species in cocoa butter and their melting and polymorphic characteristics.

TAG species	Fatty acid composition (*sn*-1/*sn*-2/*sn*-3)	Approx. melting point (°C) ⁎	Dominant polymorphic contribution	Functional relevance in chocolate
POP	Palmitate/oleate/palmitate	∼28–32	Contributes to β' → β(V) transition	Promotes early crystallization, contributes to snap and initial firmness
POS	Palmitate/oleate/stearate	∼33–36	Strongly favours β(V) polymorph	Key determinant of optimal melting range and textural balance
SOS	Stearate/oleate/stearate	∼38–42	Stabilises β(V) and β(VI) forms	Provides thermal stability, bloom resistance, and structural integrity
Minor TAGs (*e.g.* SOO, POO)⁎⁎	Mixed saturated/oleic/oleic	<28	Prefer β' forms	Excess levels lead to softness and waxy mouthfeel

Abbreviations: POP: 1,3-dipalmitoyl-2-oleoyl-glycerol; POS: 1-palmitoyl-2-oleoyl-3-stearoyl-glycerol; SOS: 1,3-distearoyl-2-oleoyl-glycerol; SOO: 1-stearoyl-2,3-dioleoyl-glycerol; POO: 1-palmitoyl-2,3-dioleoyl-glycerol.

Based on Lipp & Anklam, 1998 and Loisel et al., 1998.

⁎ Melting points refer to melting transitions of individual triacylglycerol (TAG) species, which vary depending on polymorphic form and experimental conditions.

⁎⁎ Minor TAGs are present at low abundance in cocoa butter but increase markedly in many cocoa butter substitute (CBS) formulations.

Efforts to develop cocoa butter substitute (CBS) have a long history and span multiple technological routes. Naturally occurring plant fats, including palm (*Elaeis guineensis*) mid-fraction (PMF), shea (*Vitellaria paradoxa*) butter, illipé (*Shorea macrophylla*), sal (*Shorea robusta*), and mango (*Mangifera indica*) kernel fat, have been widely adopted due to their partial compositional similarity to CB and established industrial supply chains (Jahurul et al., 2013; Lipp & Anklam, 1998) (Table 2). Chemical and enzymatic modification strategies, including fractionation and interesterification, can further reshape vegetable oils to improve compatibility with chocolate systems (Ornla-Ied, Podchong, & Sonwai, 2022; Ornla-Ied, Rungsang, et al., 2022; Zhang et al., 2020). More recently, microbial platforms have emerged as experimental routes for producing tailored fats independent of agricultural constraints (Silva et al., 2023).

**Table 2:** Comparative overview of plant-derived sources for cocoa butter substitute.

Plant source	Major TAGs	Primary functional role	Limitations	Reference
Cocoa (*Theobroma cacao*)	75–85% total SUS, including POP, POS, SOS.	Benchmark for texture, snap, and melt in chocolate	Supply volatility; high cost; susceptibility to fat bloom	Lipp & Anklam, 1998
Shea (*Vitellaria paradoxa*)	> 40% SOS, low POP; high melting point	Used as CBI, enhancing hardness and bloom resistance	Supply volatility; waxy mouthfeel	Choungo Nguekeng et al., 2021
Sal (*Shorea robusta*)	High SOS content, very high melting fat	Used as CBI to increase heat resistance	Limited supply, requires solvent fractionation	Thapa et al., 2025
Illipé (*Shorea stenoptera*)	Rich in SOS and POS, sharp melt profile	Used in premium chocolate for stable crystallization	Geographically constrained supply; high cost	Krist, 2020
Kokum (*Garcinia indica*)	POP, POS, SOS	Hardness, bloom resistance	Limited availability, local demand	Thippeswamy & Raina, 1989
Mango kernel (*Mangifera indica*)	POS, SOS, oleic-rich	Potential scale, sustainable by-product utilization	Composition varies with variety, refining challenges	Sonwai et al., 2014
*Allanblackia* spp.	SOS, stearic-rich	Sustainable, high stability	Not widely cultivated	Afoakwa, 2016
PMF	50–80% POP, low SOS, early crystallization and snap.	Dominant CBS feedstock, blending with SOS-rich fats	Sustainability concerns, low SOS limits heat stability.	Aumpai et al., 2022
HSHO sunflower stearin	Rich in symmetric SOS after fractionation	Temperate-origin.	Low crystallization low yield	Salas et al., 2023

Abbreviations: PMF: palm mid-fraction; CB: cocoa butter; SUS: saturated-unsaturated-saturated; CBI: cocoa butter improver; TAG: triacylglycerol; POP: palmitate-oleate-palmitate; POS: palmitate-oleate-saturate; SOS: stearate-oleate-stearate; PMF: palm mid-fraction; CBS: cocoa butter substitute; HSHO: high-stearic and high-oleic.

Terminology for CBS varies across the literature, reflecting differences in composition, CB compatibility, crystallization behaviour, and application. CB equivalents (CBEs) generally refer to fats that closely resemble CB in POP, POS, and SOS TAG composition and can be blended with CB without substantially altering melting or polymorphic behaviour. By contrast, CBS, CB replacers (CBRs), and CB improvers (CBIs) are often used more broadly for fats that replace or modify CB functionality in specific confectionery applications. Because this review covers diverse natural, enzymatic, microbial, and plant-biotechnological routes, we use CBS as a general umbrella term while using CBE only when referring specifically to fats with close compositional and functional equivalence to CB.

Despite progress, reproducing the functionality of CB remains a demanding challenge. Small deviations in TAG composition, stereochemistry, or crystallization behaviour can result in disproportionate losses in gloss, snap, melting profile, or bloom stability. Most existing CBS strategies therefore rely on blending, processing control, or application-specific formulations rather than true molecular equivalence. This limitation highlights the need to evaluate CBSs not only by fatty acid composition but also by TAG architecture, polymorphic stability, processing compatibility, and sustainability.

This review first establishes the molecular basis of CB functionality and defines structural and polymorphic benchmarks for CBS development. It then evaluates the major technological routes, including conventional plant-derived fats, chemical and enzymatic modification, microbial lipid synthesis, and plant metabolic engineering. By linking biochemical mechanisms with agronomic feasibility, regulatory considerations, commercial readiness, and sustainability constraints, this review aims to clarify the comparative landscape of CBS development and identify where meaningful breakthroughs are most likely to emerge.

## 2 Cocoa butter: molecular basis of composition and functionality

Cocoa butter owes its unique technological and sensory properties to an unusually constrained lipid architecture that integrates fatty acid composition, TAG stereochemistry, and polymorphic behaviour. Unlike most edible fats, CB does not derive its functionality from bulk saturation or melting point alone, but from the highly ordered assembly and crystallization of a narrow set of symmetric TAGs. Understanding this molecular logic is essential for defining realistic benchmarks for CBS and for explaining why small compositional deviations often lead to disproportionate losses in chocolate quality.

### 2.1 Origin-dependent compositional variation

Although CB is characterized by a highly conserved TAG architecture, its composition varies reproducibly with geographic origin, genotype, growing conditions, and post-harvest processing. CBs from African, Malaysian, and South American origins differ in the relative proportions of POP, POS, and SOS, producing measurable differences in firmness, melting behaviour, and crystallization properties (Ghazani & Marangoni, 2021; Lipp & Anklam, 1998). African CB is typically enriched in POS and SOS, contributing to higher melting points and greater hardness, whereas Malaysian CB contains higher POP levels and is correspondingly softer (Foubert et al., 2004). South American samples often fall between these extremes, although local genotype and environmental conditions can further modulate TAG composition and physical behaviour (Foubert et al., 2004).

These origin-specific differences are industrially important because they influence chocolate tempering, texture, bloom stability, and blending behaviour (Table 1). They also demonstrate that CB functionality exists within the relatively narrow compositional window rather than a single fixed TAG profile. For CBS development, this means that reproducing the general saturated-unsaturated-saturated (S–U–S) motif is insufficient unless the proportions of POP, POS, and SOS fall within ranges that reliably support desirable β(V) crystal formation (Alotaibi et al., 2024).

### 2.2 Triacylglycerol architecture and melting behaviour

Native CB is dominated by three TAG species, including POP, POS, and SOS, which together typically account for 80–90% of total TAGs (Jahurul et al., 2013; Lipp & Anklam, 1998; Savchina et al., 2025) (Table 1). This stereochemical uniformity enables efficient molecular packing and produces CB's sharply defined melting behaviour. The balance between palmitate, stearate, and oleate allows CB to remain solid at ambient temperature while melting rapidly near body temperature, typically between 32 and 35 °C (Jahurul et al., 2013; Lapčíková et al., 2024) (Fig. 1). Palmitic and stearic acids provide structural rigidity, whereas oleic acid at the *sn*-2 position introduces sufficient conformational flexibility to support rapid melting and flavour release in the mouth (Ramos Ramos et al., 2023). Experimental studies using structured lipids and enzymatically interesterified fats confirm that reproducing CB's melting behaviour requires not only matching fatty acid composition, but also preserving the positional distribution of fatty acids on the glycerol backbone (Singh et al., 2022).

### 2.3 Polymorphism and molecular packing

Beyond melting point, CB functionality is governed by polymorphism, the ability of TAGs to crystallize into multiple solid-state arrangements. CB exhibits at least six polymorphic forms, commonly designated forms I–VI (Fig. 1). Forms I and II are low-melting and unstable, forming rapidly during uncontrolled cooling but readily transforming into more stable structures. Forms III and IV are intermediate metastable forms that may contribute to insufficient hardness or unstable texture. Form V, also known as β(V) or β₂, is the desired polymorph in well-tempered chocolate because it provides gloss, clean snap, appropriate contraction, and resistance to fat bloom. Form VI is the most thermodynamically stable form and can develop slowly during storage, but its formation is often associated with fat bloom and quality deterioration (Ghazani & Marangoni, 2021; Loisel et al., 1998).

The ability of CB to form the desirable β(V) polymorph is closely linked to the uniform chain lengths and conserved stereochemistry of POP, POS, and SOS. These TAGs allow efficient molecular rearrangement during tempering, with saturated acyl chains at *sn*-1 and *sn*-3 stabilizing the crystal network and oleic acid at the sn-2 position supporting appropriate packing flexibility (Table 1; Yang, Lee, et al., 2024). Even modest disruption of this packing logic, such as altered POP/POS/SOS ratios, mismatched chain lengths, or randomized acyl positioning, can redirect crystallization towards less desirable β' or unstable β forms. This can result in a dull appearance, weakened texture, waxy mouthfeel, poor contraction, or accelerated fat bloom (Stobbs et al., 2025). Therefore, CBS must also be evaluated in terms of polymorphic behaviour and crystal stability. Bloom stability is usually assessed through combined fat-phase and product-level tests. Solid fat content (SFC) profiles indicate firmness at storage temperatures and residual solids near mouth temperature, while differential scanning calorimetry (DSC) tracks crystallization and melting transitions. X-ray diffraction is used to identify β' or β(I-IV), β(V)/β₂, and β(VI)/β₁ forms and monitor polymorphic transitions, and polarized light microscopy (PLM) reveals crystal size, morphology, and network structure. These fat-phase analyses are commonly complemented by accelerated storage or temperature-cycling tests, with bloom quantified by visual imaging, whiteness index, texture/hardness analysis, and sensory evaluation. Such integrated testing is essential because bloom depends not only on TAG composition and polymorphic stability but also on processing history and the non-fat matrix of chocolate (Jin & Hartel, 2020; Ramos Ramos et al., 2023).

### 2.4 Functionality of cocoa butter

The defining quality attributes of chocolate, including gloss, snap, mould release, bloom resistance, cooling sensation, and rapid melt-in-mouth, are direct consequences of CB's TAG structure and polymorphic behaviour (Widlak & Hartel, 2012). A narrow SFC profile allows chocolate to remain firm during handling and storage, while the rapid decrease in solid fat near body temperature enables smooth melting and flavour release. The β(V) crystal form contributes to surface gloss, mechanical strength, and clean fracture, whereas stable molecular packing helps delay fat bloom during storage.

Comparative functionality studies are therefore essential for determining whether a CBS or CBE can perform as a true CB alternative. Ramos Ramos et al. (2023) emphasized that fat systems mimicking CB TAG composition, particularly those dominated by POP, POS, and SOS, show the greatest potential as CBEs, whereas systems enriched in non-CB-like TAGs such as POO (1-palmitoyl-2,3-dioleoyl-glycerol) and SOO (1-stearoyl-2,3-dioleoyl-glycerol) often display altered melting behaviour, complex polymorphic transitions, or incompatibility with CB. Successful CB alternatives typically exhibit a sigmoidal SFC profile with a sharp decrease before mouth temperature, well-connected crystal microstructures, hardness and mechanical properties similar to CB, and stable β(V)/β_2_-type polymorphism (Fig. 1). These criteria provide a practical framework for comparing CBS/CBE systems with CB and for predicting their suitability in chocolate and confectionery products. These functional properties are highly sensitive to the fat phase. Small deviations in TAG composition, positional distribution, or crystallization kinetics can disrupt tempering behaviour and alter the balance between solid structure and meltability. Such changes may produce reduced snap, surface dullness, soft or waxy texture, poor demoulding, or accelerated bloom (Fig. 1).

## 3 Conventional production routes

The quest for viable CBS has long been guided by two complementary strategies rooted in physical and chemical modification. One path exploits the natural occurrence of specific plant-derived fats, such as shea or palm fractions, whose TAG compositions intrinsically mirror aspects of CB. A parallel path employs enzymatic or chemical interesterification (CIE) to deliberately reconstruct the molecular architecture of common vegetable oils. Both methodologies, despite variances in scalability and precision, strive towards the same functional benchmark: replicating the sharp melting transition and stable polymorphic form essential for high-quality chocolate.

### 3.1 Naturally derived plant fats with cocoa-butter-like TAG profiles

Naturally occurring plant fats, including palm mid-fraction (PMF), shea, illipé, sal, and mango kernel fats, remain the most widely used feedstocks for commercial CBSs (Table 2). Their intrinsic enrichment in symmetric monounsaturated TAGs, specifically POP, POS, and SOS, confers a foundational, if partial, functional compatibility with CB (Ghazani & Marangoni, 2018b; Jahurul et al., 2013; Lipp & Anklam, 1998). The utility of each fat is dictated by its dominant TAG profile; for instance, high-POP PMF promotes early crystallization and snap, while SOS-rich shea and illipé butters enhance hardness and bloom resistance (Jin et al., 2021). In practice, their functional success is often realized through strategic blending or minimal processing, such as fractionation, enabling their incorporation *via* established industrial workflows. Optimized blends, like those combining SOS-rich mango kernel fat with POP-rich PMF, can closely approximate the melting, rheological, and polymorphic behaviour of CB, allowing manufacturers to tailor the final product's functional properties (Bahari and Akoh, 2018a, Bahari and Akoh, 2018b; Sonwai et al., 2014).

Despite this maturity and utility, exotic plant fats face significant inherent constraints, as noted in Table 2. Shea butter supply is strongly influenced by seasonal and regional variability due to its reliance on semi-wild tree populations in West Africa, leading to inconsistencies in quality and availability (Choungo Nguekeng et al., 2021). Although abundant, PMF raises serious sustainability concerns linked to palm oil expansion, including deforestation, biodiversity loss, and greenhouse gas emissions (Aumpai et al., 2022). From a functional perspective, even when blended, these fats often approximate but rarely fully replicate the sharp melting transition and robust β(V) polymorphic stability of native CB.

### 3.2 Chemical and enzymatic modification of vegetable oils

To overcome the limitations of natural fats, chemical and enzymatic modification of commodity vegetable oils represents an established and scalable route to CBS. The evolution of these technologies, as detailed in Table 3, reflects a clear progression from broad, non-specific restructuring towards highly targeted molecular design. CIE was an early industrial solution, capable of randomizing fatty acids on the glycerol backbone to create fats with tailored melting curves from low-cost feedstocks like palm fractions and hydrogenated oils (Norizzah et al., 2004). However, CIE provides limited control over TAG positional distribution and generates randomized TAG structures, which restrict faithful reproduction of CB POP–POS–SOS stereochemistry. Products typically favour β' polymorphism rather than the stable β form required for tempered chocolate, limiting suitability for moulded chocolate despite good performance in coatings and fillings (Ornla-Ied, Podchong, & Sonwai, 2022; Ornla-Ied, Rungsang, et al., 2022). High processing temperatures and alkaline catalysts may also promote minor side reactions and require downstream refining.

**Table 3:** Interesterification techniques for cocoa butter substitute production.

Source	Method	Key findings/outcomes	Reference
PKS, CNO, FHPS blends	CIE with sodium methoxide catalyst	Optimal CBS from blends with 60–70% PKS, zero-*trans*, scalable.	Ornla-Ied, Podchong, & Sonwai, 2022; Ornla-Ied, Rungsang, et al., 2022
Palm stearin & palm kernel olein blends	CIE	With β’-tending polymorph, suitable for coatings and fillings, limited stereochemical precision and stable β polymorph formation, limited use in tempered chocolate.	Norizzah et al., 2004
Palm oil & hydrogenated soybean oil	1,3-specific *Rhizomucor miehei* lipase	45% yield of CBS *via* ester-ester exchange	Abigor et al., 2003
PMF & stearic acid	1,3-specific *R. miehei* lipase	Maintaining *sn*-2 oleic acid and enriching SOS/POS-type TAGs	Ibrahim, 2013; Huang et al., 2021
Palm olein & PFAD (stearic/palmitic acids)	1,3-specific *R. miehei* lipase	Used refinery by-product PFAD to synthesize CBS, adding value to waste streams.	Ibrahim, 2012
PMF & stearic acid	1,3-specific *Rhizopus arrhizus* lipase	Early demonstration of enzymatic CBS production, including process intensification in a gas-lift reactor.	Mojović, Šiler-Marinković, Bugarski, et al., 1994, Mojović, Šiler-Marinković and Knežević, 1993
Palm oil & methyl stearate	1,3-specific *Carica papaya* (papaya) lipase	55% yield of CBS using a plant-based lipase, offering a non-microbial enzymatic alternative.	Pinyaphong & Phutrakul, 2009
PMF & fully hydrogenated soybean oil	1,3-specific *Thermomyces lanuginose* lipase	20.5% yield of CBS, using fully hydrogenated oils in EIE.	Soekopitojo et al., 2009
Palm oil & tristearin	1,3-specific *R. miehei* lipase in supercritical CO₂	53% yield of CBS, showcasing green process engineering.	Liu et al., 1997
Palm olein & stearic acid	1,3-specific *R. miehei* lipase, followed by fractionation	Demonstrating that EIE + fractionation hybrid process	Chong et al., 1992
PMF, PKO, palm stearin blend & stearic/oleic acids	1,3-specific *T. lanuginose* lipase	Compatible with real CB in blends	Biswas et al., 2018
PMF, PKO, & MCT oil	1,3-specific lipase	Low-calorie CBS by incorporating MCTs	Borhan et al., 2011
PMF & stearic acid	1,3-specific commercial lipase (Lypozyme)	Direct and scalable	Undurraga et al., 2001
Camel hump fat and tristearin	EIE in supercritical CO_2_, optimized by RSM	Improved yield by optimizing temperature and pressure	Shekarchizadeh et al., 2014

Abbreviations: CB: cocoa butter; CIE: chemical interesterification; PKS: palm kernel stearin; CNO: coconut oil; FHPS: fully hydrogenated palm stearin; EIE: enzymatic interesterification; PMF: palm mid fraction; PKO: palm kernel oil; PFAD: palm fatty acid distillate; MCT: medium-chain triacylglycerols; RSM: response surface methodology.

In contrast, enzymatic interesterification (EIE) using *sn*-1,3-specific lipases has become the preferred strategy for creating true CBSs (Table 3). This method allows for the selective restructuring or direct synthesis of target SOS-type TAGs, preserving the stereochemistry critical for CB-like crystallization and polymorphic stability (Ghazani & Marangoni, 2018a; Sivakanthan & Madhujith, 2020). The most significant recent advance is the integration of EIE with physical fractionation. For example, applying EIE to high-stearic (HS)/high-oleic (HO) sunflower oil *prior* to dry fractionation substantially modifies its crystallization behaviour, thereby overcoming key limitations of the native oil (Salas et al., 2023). EIE creates a more favourable, randomized TAG profile, which allows a more efficient and higher-yielding fractionation without the need for dewaxing or seeding, resulting in a superior CBS from a temperate oilseed (de Figueiredo et al., 2025; Marangoni & McGauley, 2003). Complementary standalone fractionation strategies, such as the multi-stage solvent fractionation of mahua oil to enrich POS (Thilakarathna et al., 2024), remain crucial for refining TAG composition from specific sources. However, the synergy of EIE and fractionation defines the current state of the art, maximizing both yield and functional fidelity. Overall, enzymatic and combined modification routes deliver high functional performance and industrial applicability (Liu et al., 1997; Zhang et al., 2020). Yet, they remain constrained by processing costs and complex optimisation. These persistent challenges, alongside regulatory landscapes (Mokbul & Siow, 2022), underscore the compelling motivation for upstream biotechnological solutions that aim to produce CB-like TAGs *de novo* within biological organisms.

## 4 Microbial synthesis: from mechanistic models to engineered cell factories

Oleaginous microorganisms have emerged as promising alternative platforms for CBS synthesis, driven by their high lipid productivity, rapid growth cycles, and capacity for industrial-scale fermentation (Ratledge, 2002). Yeasts such as *Saccharomyces cerevisiae*, *Yarrowia lipolytica*, and *Rhodotorula toruloides* can accumulate substantial lipid stores (20–70% of dry cell weight) and naturally synthesize the requisite C16 and C18 fatty acids (Ledesma-Amaro & Nicaud, 2016). However, native microbial lipidomes are dominated by TAGs with diverse and often asymmetric structures. In *S. cerevisiae*, lipid profiles are typically enriched in palmitoleic acid (C16:1^Δ9^), whereas many oleaginous yeast species accumulate high levels of polyunsaturated fatty acids. As a result, SOS-type TAGs are rare or absent in microbial systems (Wei, Gossing, et al., 2017; Wei, Siewers, & Nielsen, 2017). Consequently, microbial CBS production necessitates extensive metabolic engineering to simultaneously boost total lipid yield and rewire TAG assembly for stereochemical specificity (Wang et al., 2020).

### 4.1 Engineering strategies and key metabolic insights

**Fig. 2:**
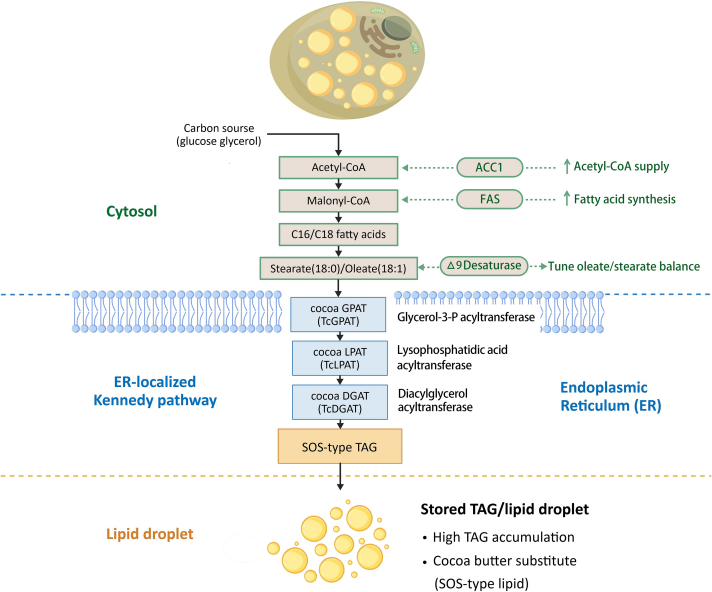
Engineering oleaginous yeasts for cocoa butter substitute lipid production. The figure illustrates the metabolic route by which oleaginous yeasts can be engineered to produce cocoa butter-like TAGs. Carbon sources such as glucose or glycerol are converted in the cytosol into acetyl-CoA and then malonyl-CoA. Overexpression of targets genes such as *ACC1* and *FAS* increase acetyl-CoA supply and fatty acid synthesis, generating C16/C18 fatty acid substrates. Δ9 desaturase activity is adjusted to tune the balance between stearate (C18:0) and oleate (C18:1^Δ9^), which are the key acyl chains required for SOS-type TAG formation. At the ER, the Kennedy pathway sequentially assembles TAGs through cocoa-derived GPAT, LPAT, and DGAT enzymes, which promote incorporation of stearate and oleate into cocoa butter-like TAG structures. The resulting SOS-type TAGs are then stored in lipid droplets, enabling high TAG accumulation and providing a microbial route for CBS production. Abbreviations: TAG, triacylglycerol; ACC1, acetyl-CoA carboxylase; CBS, cocoa butter substitute; DGAT, diacylglycerol acyltransferase; ER, endoplasmic reticulum; FAS, fatty acid synthase; GPAT, glycerol-3-phosphate acyltransferase; LPAT, lysophosphatidic acid acyltransferase; SOS-type TAG, triacylglycerols enriched in stearoyl–oleoyl–stearoyl-type structures.

Microbial CBS/CBE engineering follows a two-step strategy (Fig. 2). First, it expands the precursor pools of stearate and oleate while reducing unwanted fatty acids like palmitoleic acid. Second, it introduces or modifies acyltransferases to enforce SOS-type assembly. In *S. cerevisiae*, precursor engineering has targeted key metabolic nodes (Table 4). Overexpression of a deregulated *Acetyl-CoA Carboxylase* (*ACC1*) increases malonyl-CoA flux (Wang et al., 2016). Modulating elongases (*ELO1*, *ELO2*) and the Δ9-desaturase (*OLE1*) shifts the profile towards stearic and oleic acids (Bergenholm et al., 2018). Multi-omics analyses of cocoa and shea enabled the mining of genes encoding glycerol-3-phosphate acyltransferase (GPAT), lysophosphatidic acid acyltransferase (LPAT), and diacylglycerol acyltransferase (DGAT) that are implicated in CB TAG assembly (Wei et al., 2018; Wei, Gossing, et al., 2017). These enzymes function sequentially within the Kennedy pathway, in which GPAT, LPAT, and DGAT catalyze acyl-CoA esterification at the *sn*-1, *sn*-2, and *sn*-3 positions of the glycerol backbone, respectively (Lung & Weselake, 2006). Functional expression of these plant-derived acyltransferases in yeast systems demonstrated a markedly enhanced capacity to assemble CB-like TAGs. In one study, combinatorial expression of cocoa *GPAT*, *LPAT*, and *DGAT* in an engineered *S. cerevisiae* strain increased total TAG 134-fold and significantly raised SOS-type lipids (Wei et al., 2018). A foundational platform for engineering yeast as a microbial factory was established by genetically reprogramming *S. cerevisiae* strains from ethanol fermentation to high-yield lipogenesis, enabling fatty acid production for CBS applications. This approach, as noted in Table 4, integrating pathway engineering, process optimization, and adaptive evolution with pyruvate kinase mutations, resulted in extracellular free fatty acid titres reaching 33.4 g/L (Yu et al., 2018).

**Table 4:** Microbial production of cocoa butter substitute.

host organism	Genetic modification strategy	Key outcomes	Significance	References
*Saccharomyces cerevisiae*	Expression of codon-optimized cocoa (*Theobroma cacao*) acyltransferase genes, *GPAT, LPAT,* and *DGAT*	• TAG increased by 2.25-fold • POP, POS, SOS increased by 190–230% • CBS production increased by 6.7-fold	Proof-of-concept for a fermentation-based microbial CBS production.	Wei, Gossing, et al., 2017
*S. cerevisiae*	Expression of *GPAT, LPAT,* and *DGAT* in a mutant strain (*IMX581 sct1Δ ale1Δ lro1Δ dga1Δ*) with a disrupted native TAG pathway	• 8-fold CBS increase in SYJ-441 • 134-fold CBS increase in Y29-TcD1 • Total TAG increase	Evaluated the feasibility of engineering yeast as cell factories for sustainable CBS production.	Wei et al., 2018
Six yeast species: *S. cerevisiae, Trichosporon oleaginosus*, *Rhodotorula graminis*, *Lipomyces starkeyi*, *Rhodosporidium toruloides*, *Yarrowia lipolytica*	• Comparative cultivation in nitrogen-limited medium to induce lipid accumulation. • No genetic modifications	• *T. oleaginosus* produced the most TAGs at 378 mg/g DCW. • *T. oleaginosus* produced 27.8% POP+POS content. • < 3% SOS in all yeasts.	Identified *T. oleaginosus* as the most promising natural producer of CBS lipids among the tested yeasts.	Wei, Siewers, & Nielsen, 2017
*S. cerevisiae*	• Engineered the *ACC1* variant (*ACC1S659A S1157A* / *ACC1*) to abolish *Snf1* regulation. • Combined overexpression of *ACC1, OLE1* (Δ9-desaturase), and *ELO1* (elongase).	• TAG increased by 5.8-fold; SOS increased by 48-fold. • Increased C18 fatty acid composition; decreased palmitoleic acid (C16:1).	Coordinated engineering of precursor supply, desaturation, and elongation as a strategy to enhance CBS in yeast.	Bergenholm et al., 2018
*S. cerevisiae*	• Transcriptomic analysis of shea fruits (*Vitellaria paradoxa*) to identify TAG biosynthetic genes. • Functional validation in both wild-type and TAG-pathway-deficient yeast strains.	• Identified 14 shea TAG biosynthetic genes (*GPATs, LPATs, DGATs*). • *VpDGAT1* and *VpDGAT7* restored TAG production in a DGAT-deficient strain. • Expression of shea genes altered yeast lipid profiles**,** though SOS production remained low in single-gene expression strains.	Expressing plant TAG biosynthetic genes in yeast, expanding the toolkit for microbial production of CBS lipids**.**	Wei et al., 2019
*Apiotrichum brassicae*	• Selected from 11 oleaginous yeast strains. • Cultivated on dairy permeates in lab • Pilot-scale • Without genetic engineering.	• Produced ∼31% stearic acid when grown on glucose/galactose-rich side stream. • TAG profile contained POP, POS, SOS at similar ratios. • High-melting similar to non-tempered cocoa butter.	• Demonstrates the potential of using specific fermentation conditions to produce CBS. • CBS production using industrial side streams.	Småros et al., 2025
*Y. lipolytica*	Replacing native Δ9 desaturase (*Ole1p*) with homolog from *R. toruloides* (*RTOLE1*); wild-type Δ12 desaturase (FAD2) retained.	Generated optimal fatty acid profile close to desired CBSs: with 26% palmitic acid, 24% stearic acid, 42% oleic acid, and 8% linoleic acid	• Increased lipid content ∼45% of DCW; • Growth unaffected.	Konzock et al., 2022
*Y. lipolytica*	Engineered with high-lipid background (ARE1 deletion, DGA1 overexpression); native desaturases.	Increased stearic acid and reduced linoleic acid.	2.5-fold increase in lipid content.	Konzock et al., 2022
*R. toruloides*	Optimization of fermentation conditions (carbon source, nitrogen source, Mg^2+^, pH).	• Max biomass: 7.8 g/L • Max lipid content: 61.0% • Max lipid production: 4.4 g/L • Oil composition mimics CB. • POP, POS, SOS constituted 27.11% of total TAGs	• Proof-of-concept for fermentative CBS production. • Scalable, and non-seasonal alternative to traditional CB sourcing.	Yang, Lu, et al., 2024

Abbreviations: TAG: triacylglycerol; CBS: cocoa butter substitute; SOS: 1,3-distearoyl-2-oleoyl-glycerol; POS: 1-palmitoyl-2-oleoyl-3-stearoyl-glycerol; POP: 1,3-dipalmitoyl-2-oleoyl-glycerol; GPAT: glycerol-3-phosphate acyltransferase; LPAT: lysophosphatidic acid acyltransferase; DGAT: diacylglycerol acyltransferase; DCW: dry cell weight; ACC: acetyl-CoA carboxylase; OLE1: yeast Δ9-desaturase; ELO1: fatty acid elongase 1.

### 4.2 Recent progress and shift towards oleaginous yeasts

Recent work has shifted from *S. cerevisiae* towards native oleaginous yeasts with higher intrinsic lipogenic capacity. Species such as *Y. lipolytica* and *R. toruloides* can accumulate high lipid levels, often 40–65% of dry cell weight, possess abundant acetyl-CoA pools, and have increasingly mature genetic tools, making them attractive chassis for CBS production (Abeln & Chuck, 2021). In *Y. lipolytica*, desaturase engineering has reduced palmitoleic and linoleic acids and generated fatty acid profiles closer to CB while maintaining relatively high lipid accumulation in selected backgrounds (Konzock et al., 2022). *Rhodotorula toruloides* has also been engineered to accumulate 55–60% lipid content with CBS-relevant profiles under nitrogen limitation (Yang, Lu, et al., 2024). *Cutaneotrichosporon oleaginosus* is another promising host, naturally accumulating up to 43% lipids and approximately 28% CB-like lipids under nitrogen limitation (Wei, Siewers, & Nielsen, 2017; Table 4). This shift to oleaginous yeasts therefore improves the biological feasibility of microbial CBS production by combining higher lipid accumulation with greater metabolic flexibility.

However, microbial oils must be evaluated not only by lipid titre or total stearate/oleate content, but by whether these fatty acids are assembled into CB-like TAG structures. Increasing stearate and oleate pools can improve the saturated/monounsaturated balance required for sharp melting, while heterologous expression of cocoa-derived *GPAT*, *LPAT*, and *DGAT*, as demonstrated by Wei, Gossing, et al. (2017)), Wei, Siewers, and Nielsen (2017), and Wei et al. (2018), provides a route to enrich SOS-type TAGs with greater positional specificity. Because TAG composition governs crystallization, SFC, polymorphic behaviour, and CB compatibility, oils enriched in non-CB-like or low-melting TAGs such as POO and SOO may still show altered melting behaviour, eutectic incompatibility, or increased bloom risk (Ramos Ramos et al., 2023). Therefore, microbial CBS development must progress from compositional engineering alone towards integrated validation of melting profile, β(V)-type crystallization, tempering behaviour, and bloom stability under chocolate-processing conditions.

Despite this progress, commercial feasibility remains constrained by titre, productivity, substrate cost, and food-grade process compatibility. In *S. cerevisiae*, low native lipid accumulation and negligible SOS-type TAG formation require extensive engineering before industrial productivity can be approached (Wang et al., 2020). Even in oleaginous yeasts, compositional precision may impose productivity penalties; for example, stronger reduction of linoleic acid in engineered *Y. lipolytica* generated CB-like fatty acid profiles but reduced biomass from 18 to 10 g/L and lipid content from 45% to 20% in one engineered background (Konzock et al., 2022). Thus, microbial platforms provide programmable routes to CB-like lipids, but their viability will depend on achieving high titres of correctly structured TAGs on low-cost substrates while maintaining productivity and food-grade processing compatibility.

### 4.3 Economic, downstream-processing, and food-grade constraints

Microbial CBS production also faces downstream-processing and food-grade compliance challenges. Unlike plant seed oils, microbial oils are intracellular and require biomass harvesting, cell disruption, lipid extraction, purification, refining, and sensory-quality validation. Oleaginous yeasts can accumulate substantial intracellular lipid reserves, but recovery efficiency depends on disrupting robust yeast cell walls using thermal, acid/alkaline, enzymatic, ultrasonication, bead-milling, or high-pressure homogenization approaches (Nguyen et al., 2025). Reported examples include acidification with 2.0–2.5 M HCl for 30 min at 100 °C, ultrasonication-assisted solvent extraction, and sonication followed by β-glucanase/mannanase treatment, which achieved 95.4% lipid recovery from a 10% biomass suspension in one process (Nguyen et al., 2025). However, many laboratory-scale protocols still use chloroform/methanol or similar solvents, highlighting the need for scalable food-compatible extraction and solvent-recovery systems. Extracted oils must then be refined and deodorized to remove residual biomass, phospholipids, free fatty acids, pigments, sterols, oxidation products, residual solvents, and fermentation-derived volatiles while preserving the CB-like TAG profile required for sharp melting and stable crystallization. Food-grade use also requires toxicological evaluation, strain safety documentation, assessment of antibiotic-resistance or toxic-metabolite risks, and regulatory approval, particularly when genetically modified strains are used (Nguyen et al., 2025). Thus, microbial CBS feasibility depends not only on engineering high lipid titre and SOS-type TAG accumulation, but also on reducing extraction, purification, deodorization, and compliance costs.

### 4.4 Commercial landscape and future outlook

Despite compelling scientific progress, the commercial production of microbially derived CBS remains nascent, positioned primarily at the pilot or pre-commercial stage. The primary barriers are economic, relating to the high cost of fermentation feedstocks, downstream lipid extraction, and purification to food-grade standards, coupled with regulatory pathways for novel food ingredients that are lengthy and complex (Abeln & Chuck, 2021). Nevertheless, pioneering startups are beginning to translate this research into commercial ventures. Companies like C16 Biosciences are developing yeast-based platforms to produce sustainable alternatives to palm oil. While their initial commercial targets are often in cosmetics or general-purpose oils, the underlying technology for tailoring yeast lipid profiles provides a direct pathway towards CBS. These ventures illustrate growing industrial interest in microbial oil synthesis, although dedicated CBS production for chocolate remains a longer-term application.

Overall, engineered microbial platforms have transitioned from pure mechanistic models to advanced proof-of-concept cell factories. Their most significant impact to date has been deconvoluting the enzymatic basis of SOS synthesis, offering critical insights into TAG assembly logic. While not yet cost-competitive with large-scale plant-derived CBS, continued advances in strain engineering, fermentation technology, and supportive regulatory frameworks may position microbial synthesis as a viable source for high-value, specialized lipid ingredients in the future. These foundational insights from microbial systems also inform a complementary and fundamentally distinct production strategy, which is the direct *in planta* engineering of oilseed crops.

## 5 Plant biotechnology strategies for cocoa butter substitutes

Plant biotechnology provides a fundamentally distinct route to CBS by enabling direct manipulation of fatty acid biosynthesis and TAG assembly within developing seeds. Unlike blending or post-harvest modification, this approach seeks to reconstruct CB-like lipid structures *in planta*, targeting both the supply of palmitic, stearic, and oleic acids and their ordered incorporation into POP, POS, and SOS TAGs. Decades of research on seed oil metabolism have identified key enzymatic control points governing these processes, forming the foundation for current CBS engineering strategies (Bates et al., 2013; Bates & Shockey, 2025; Napier et al., 2014).

### 5.1 Enzymatic control points in fatty acid biosynthesis and triacylglycerol assembly

Fatty acid composition in seed oils is established primarily in plastids, where chain length and saturation are regulated by the fatty acid synthase (FAS) complex. The balance between palmitate and stearate is determined by competition between β-ketoacyl-ACP synthase II (KASII), which elongates palmitoyl-ACP to stearoyl-ACP, and acyl-ACP thioesterases such as FatB, which terminate elongation by hydrolysing palmitoyl-ACP (Salas & Ohlrogge, 2002). Stearate accumulation is further constrained by stearoyl-ACP desaturase (SAD), which rapidly converts stearoyl-ACP to oleate, making SAD a major gatekeeper for saturated C18 fatty acid levels (Mustiga et al., 2019). In the endoplasmic reticulum (ER), fatty acid desaturase 2 (FAD2) catalyzes the conversion of oleate to linoleate, exerting strong control over the monounsaturated–polyunsaturated balance in storage lipids (Nguyen et al., 2019). TAG assembly enzymes, including GPAT, LPAT, and DGAT, exhibit intrinsic substrate preferences that favour unsaturated acyl-CoAs in most temperate oilseeds, biasing TAG composition towards polyunsaturated species (Liao et al., 2022; Lung & Weselake, 2006). Because CB functionality depends on oleic acid at the *sn*-2 position and saturated fatty acids at *sn*-1 and *sn*-3, achieving CB-like TAG profiles requires coordinated manipulation of both fatty acid biosynthesis and acyltransferase specificity.

### 5.2 Engineering fatty acid pools: high-oleic, high-stearic, and high-palmitic traits

As summarized in Table 5, plant biotechnology has repeatedly demonstrated that the major fatty-acid components underpinning CB functionality, including oleic, stearic, and palmitic acids, can each be manipulated effectively in oilseed crops. Over several decades, an extensive body of research has generated HO oils across numerous crop species using diverse genetic strategies and regulatory designs. By comparison, substantially fewer studies have focused on the development of HS and high-palmitic (HP) phenotypes. Owing to the scale of the HO literature and the more limited but growing work on HS and HP oils, only representative and mechanistically informative examples are highlighted here (Table 5).

**Table 5:** Plant mutants and genetic engineering strategies targeting CBS fatty acid profiles.

Host species	Target trait	Genetic modification strategy	Key lipid outcome	Relevance to CBS	Limitation	Reference
*Helianthus annuus*	HO	Chemical mutagenesis	>70% oleate	Supplies oleate pool required for *sn*-2 position	Lacks sufficient saturated FA; random TAG assembly	Soldatov, 1976
*H. annuus*	HS	HS mutant CAS-3, generated *via* EMS chemical mutagenesis	25% stearate	Approaches SOS-like saturation	Poor germination; cold sensitivity	Osorio et al., 1995
*H. annuus*	HP	HP mutant CAS-5, generated *via* X-ray mutagenesis; HP + HO mutant CAS-12, generated *via* X-ray mutagenesis of Pervenets-type HO mutant BSD-2-423.	CAS-5: 33% palmitate; CAS-12: 30% palmitate, 55% oleate, and 7% palmitoleate	The combination of HP and HS was not possible, as HP loci showed an epistatic effect over HS loci.	Reduced plant height, leaf number, low yield	Pérez-Vich et al., 1999; Pérez-Vich et al., 2002.
*Glycinemax*	HO	*FAD2–1 A*/*FAD2–1B* silencing (RNAi, genome editing)	75–80% oleate	Industrially scalable HO platform	CB requires high SFA at *sn*-1/3	Pham et al., 2010; Demorest et al., 2016
*Brassica napus*	HS	Seed-specific *SAD* antisense	20–25% stearate	Supplies stearate required for SOS	Growth penalties if expression not tightly controlled	Knutzon et al., 1992
*G. max*	HS + HO	Mangosteen stearoyl-ACP thioesterase + *FAD2* suppression	∼19% stearate, ∼70% oleate	Partial FA-level mimicry of CB	Oleate unstable under field conditions	Hawkins & Kridl, 1998; Park et al., 2014
*Gossypium hirsutum*	HS + HO	*SAD* + *FAD2–1* RNAi	∼30–40% stearate, ∼38–45% oleate	Closest FA-level match to CB	Palmitate too low; TAG structure incorrect	Liu et al., 2002
*A. thaliana*	HP	KASII loss-of-function	>40% palmitic acid	Supplies palmitate for POP/POS	Severe membrane effects	Falcone et al., 2004
*G. hirsutum*	HP	Seed-specific *KASII* RNAi	50–53% palmitate	Palm-like palmitate supply	Excess palmitate disrupts fatty acid balance	Liu et al., 2017
*G. hirsutum*	HP + HO	*KASII* RNAi × *FAD2–1* suppression	High palmitate + oleate	Theoretically POP-favourable	Stearate reduced	Liu et al., 2017
*G. hirsutum*	HP + HS	*KASII* RNAi × *SAD* suppression	Palmitate dominant; stearate reduced	Demonstrates metabolic hierarchy	HP overrides HS; non-additive	Liu et al., 2017
*B. napus*	FA pool + TAG assembly	*DGAT*/*LPAT* overexpression	Altered TAG spectrum	Shows need for assembly control	No SUS stereochemistry	Liao et al., 2022

Abbreviations: TAG: triacylglycerols; FA: fatty acids; CB: cocoa butter; CBS: cocoa butter substitute; SAD: stearoyl-ACP desaturase; FAD2: omega-6 oleoyl-PC desaturase; KASII: keto acyl synthase II; DGAT: diacylglycerol acyltransferase; LPAT: lysophosphatidic acid acyltransferase; POS: 1-palmitoyl-2-oleoyl-3-stearoyl-glycerol; POP: 1,3-dipalmitoyl-2-oleoyl-glycerol; SOS: 1,3-distearoyl-2-oleoyl-glycerol; SUS: saturated-unsaturated-saturated; SFA: saturated fatty acid; HP: high-palmitic; HS: high-stearic; HO: high-oleic.

Among these traits, HO oils are the most mature and widely implemented outcome of seed-lipid engineering. Natural and induced mutations in *FAD2*, together with antisense, RNAi, and more recently genome-editing approaches, reliably elevate oleic acid content above 70% in a broad range of species, including sunflower, safflower, soybean, rapeseed, cotton, and camelina (Demorest et al., 2016; Knowles & Hill, 1964; Liu et al., 2002; Pham et al., 2010; Soldatov, 1976; Taliercio et al., 2025). Commercial deployment of HO soybean oils illustrates the agronomic stability and industrial scalability of this modification (Damude & Kinney, 2008; Gangurde et al., 2025). From a CBS perspective, HO traits provide a robust oleate pool compatible with the *sn*-2 position of CB TAGs; however, HO oils alone lack the saturated fatty acid supply required to support POP, POS, and SOS formation.

Increasing stearic acid content has proven more challenging due to the essential role of unsaturated fatty acids in membrane function. Early HS sunflower mutants revealed strong temperature sensitivity and poor germination, highlighting the physiological penalties associated with excessive stearate accumulation (Fernández-Moya et al., 2002; Salas, Bootello, Martínez-Force, et al., 2011, Salas, Martínez-Force, Harwood, et al., 2014). HS mutants in soybean were also reported to cause pleiotropic changes in somatic tissues (Carrero-Colón et al., 2014; Gillman et al., 2014). SAD converts stearoyl-ACP to oleoyl-ACP, helping maintain the saturated/monounsaturated fatty acid balance required for membrane fluidity, seed development, and stress adaptation (Fernández-Moya et al., 2002; Liu et al., 2002). Excessive suppression or mutation of *SAD* may therefore increase stearate in storage TAGs and, depending on tissue specificity, in membrane lipid pools, reducing membrane fluidity, altering lipid phase behaviour, and impairing germination or seedling establishment, particularly under low-temperature conditions (Dhaliwal et al., 2024).

Seed-specific suppression of *SAD* partially alleviates these effects and enables substantial stearate enrichment in *Brassica* and cotton (Knutzon et al., 1992; Liu et al., 2002). Complementary approaches exploiting rare stearoyl-ACP thioesterases, such as those from mangosteen, further enhance stearate export from plastids (Hawkins & Kridl, 1998). In soybean, combining a mangosteen thioesterase with silencing of palmitoyl-ACP thioesterase (*FatA/B*) and *FAD2* produced oils containing approximately 19% stearate and about 70% oleate, with the phenotype stable across generations but sensitive to field conditions (Park et al., 2014). In cottonseed, simultaneous suppression of *SAD* and *FAD2* yielded oils with approximately 30–40% stearic acid and about 38–45% oleic acid, approaching the saturated-to-monounsaturated balance of CB at the fatty-acid level (Liu et al., 2002). Notably, these HS/HO cotton lines exhibited reduced palmitic acid content, limiting their capacity to reconstruct CB-like TAG profiles.

In sunflower, the HP mutant CAS-5, generated *via* X-ray mutagenesis, contains approximately 25–33% palmitic acid, although elevated palmitate is insufficient to confer CB-like functionality (Table 5; Osorio et al., 1995; Pérez-Vich et al., 1999). CAS-12, derived from the Pervenets-type HO background, combines about 30% palmitate with 55% oleate, making it more relevant for POP/POS-rich fat design (Pérez-Vich et al., 2002). Mechanistically, palmitic acid accumulation is primarily governed by the balance between KASII-mediated elongation of palmitoyl-ACP to stearoyl-ACP and acyl-ACP thioesterase activity. Partial loss of function or seed-specific downregulation of *KASII* can markedly increase palmitate accumulation, as shown in *Arabidopsis* and cotton (Falcone et al., 2004; Liu et al., 2017; Pidkowich et al., 2007). In cotton, *KASII* RNAi lines accumulated approximately 50–53% palmitic acid, approaching palm-oil-like compositions (Liu et al., 2017). When HP traits were stacked with HO backgrounds, palmitate and oleate accumulated effectively, suggesting compatibility with POP-rich TAG formation. In contrast, stacking HP with HS traits produced strongly non-additive outcomes, with stearate accumulation greatly reduced while palmitate remained dominant (Liu et al., 2017). This agrees with sunflower crosses between CAS-3 and CAS-5, where HP and HS phenotypes could not be fully combined because HP loci epistatically suppressed expression of the HS trait, limiting the development of sunflower oil simultaneously enriched in both palmitic and stearic acids (Pérez-Vich et al., 2002). Overall, these results suggest asymmetric competition for shared acyl-ACP pools and a metabolic hierarchy in which HP traits tend to override HS accumulation, likely due to preferential removal of palmitoyl-ACP by FatB and reduced substrate availability for KASII-dependent stearate formation.

Collectively, the studies summarized in Table 5 demonstrate that HO, HS, and HP traits are individually achievable and, when appropriately confined to seeds, agronomically viable. However, attempts to combine these traits reveal strong metabolic constraints and non-additive interactions that prevent faithful reconstruction of the balanced palmitate-stearate-oleate composition of CB. Even in lines where fatty acid compositions approximate that of CB, the resulting TAG species remain structurally divergent, underscoring that fatty acid engineering alone is insufficient to generate functional CBS.

### 5.3 Engineering TAG assembly and enforcing POP–POS–SOS stereochemistry

Cocoa butter is defined by the highly ordered assembly of palmitate, oleate, and stearate into POP, POS, and SOS TAGs. In cocoa seeds, this molecular architecture does not arise solely from fatty acid composition but is actively imposed by acyltransferases with strong positional selectivity (Bates & Shockey, 2025; Napier et al., 2014). Transcriptomic analyses of developing *T. cacao* seeds reveal coordinated upregulation of GPAT, LPAT, and DGAT isoforms during the phase of rapid TAG accumulation, coinciding with enrichment of saturated and monounsaturated fatty acids (Li et al., 2019). Earlier isotope-labelling studies further demonstrated that cocoa GPAT preferentially incorporates palmitate at the *sn*-1 position, LPAT exhibits strong specificity for oleate at *sn*-2, and DGAT efficiently accepts stearate at the *sn*-3 position, collectively enforcing the conserved saturated–unsaturated–saturated (S–U–S) TAG architecture characteristic of CB (Griffiths & Harwood, 1991). Temperate oilseeds generally lack this level of stereochemical control. Even when fatty acid pools are engineered towards CB-like compositions, endogenous LPAT and DGAT isoforms exhibit broad substrate tolerance and preferentially incorporate unsaturated acyl-CoAs, resulting in heterogeneous and asymmetrically substituted TAG species with inappropriate melting and polymorphic behaviour (van Erp et al., 2019). This limitation explains why plant lines with fatty acid profiles approaching CB still fail to reproduce its crystallization behaviour and functional performance. Introducing cocoa- or shea-derived acyltransferases therefore represents a rational strategy for imposing POP–POS–SOS architecture in heterologous hosts, but success has so far been limited.

Insight into this limitation can be gained by comparison with engineered yeast systems, in which TAG stereochemistry has proven substantially more tractable. In *S. cerevisiae* and oleaginous yeasts, heterologous expression of cocoa- or shea-derived GPAT, LPAT, and DGAT isoforms has enabled significant enrichment of SOS-type TAGs, even when precursor fatty acid pools remain imperfect (Konzock et al., 2022; Wei et al., 2018). These outcomes reflect fundamental differences in lipid metabolic organization between microbial and plant systems. In yeasts, TAG biosynthesis is largely uncoupled from membrane lipid homeostasis, allowing substantial remodeling of storage lipid stereochemistry without catastrophic effects on cellular viability. In contrast, plant fatty acid and TAG biosynthesis are tightly integrated with membrane formation during seed development, such that perturbations in acyltransferase specificity or fatty acid saturation readily spill over into phospholipid pools, imposing strong physiological constraints (Bates et al., 2013).

Spatial and regulatory complexity further amplifies this disparity. TAG assembly in developing plant seeds is distributed across plastids, ER subdomains, and lipid droplets, with strong developmental and tissue-specific regulation. Endogenous acyltransferases are abundant and highly competitive, making it difficult for introduced enzymes to impose positional selectivity unless expression levels, timing, and subcellular localization are precisely matched to native TAG biosynthesis. By contrast, yeast TAG synthesis is spatially simpler and temporally flexible, enabling heterologous acyltransferases to exert disproportionate control over TAG composition once expressed at sufficient levels (Konzock et al., 2022; Wei et al., 2018).

Together, these differences explain why plant-based CBS efforts have struggled to replicate CB-like stereochemistry despite decades of success in fatty acid engineering. They also clarify why modifying acyltransferase identity alone is insufficient *in planta*. Achieving faithful POP–POS–SOS assembly in oilseed crops will require coordinated control of acyltransferase competition, acyl-CoA pool partitioning, and subcellular targeting, alongside careful management of membrane lipid homeostasis. This systems-level challenge represents a central bottleneck for plant-derived CBS and underscores the need for integrated metabolic and regulatory engineering strategies.

### 5.4 Candidate crop platforms and proof-of-concept efforts

The selection of a host organism for CBS engineering presents a fundamental and unresolved trade-off between inherent metabolic compatibility and agricultural scalability. This choice dictates the scale of the required genetic intervention and the likelihood of pleiotropic side effects, defining the core strategic challenge for the field. On one side of this dilemma are tropical tree species such as shea, sal, and *Allanblackia spp.* These species are metabolically pre-adapted, naturally accumulating S-U-S-rich TAG profiles that closely mirror CB (Afoakwa, 2016; Lipp & Anklam, 1998). As such, they represent valuable biological models for understanding CB-like lipid assembly. However, their agronomic profiles are prohibitive for scalable production. They exhibit long juvenile phases, irregular yields, restricted cultivation geographies, and a general paucity of genomic and transformation resources. These limitations render them unsuitable as near-term, engineered platforms for meeting global demand.

While high-yielding temperate oilseeds like soybean, rapeseed, cotton, and camelina offer exceptional agronomic scalability (Liu et al., 2002; Okuzaki et al., 2018), they remain a recalcitrant chassis for CB production. These crops possess well-established genetic toolkits, and traits for elevated stearate or palmitate are demonstrable (Liu et al., 2017). Yet, their physiology is fundamentally misaligned with the CB phenotype. The lipid metabolism in these temperate oilseed species is optimized for polyunsaturated oils, and their native acyltransferases lack the stereospecificity for POP-POS-SOS assembly (Pham et al., 2010). Crucially, engineering a high-saturate supply risks pleiotropic effects, as non-native fatty acids can incorporate into membrane lipids. This disrupts cellular physiology, rendering plants more sensitive to cold and often impairing germination, which is a critical vulnerability in temperate climates (Liu et al., 2002). Consequently, despite decades of fatty acid engineering, no temperate oilseed has been engineered to produce an authentic CB TAG profile. This persistent gap underscores that altering substrate supply is necessary but insufficient; the decisive step is the concomitant and far more delicate engineering of TAG assembly logic without compromising the host's agronomic fitness. The core biotechnological challenge is therefore to bridge this divide by imparting precise biosynthetic machinery into a scalable host, a systems-level endeavour that must succeed not only in the laboratory but also within the complex realities of supply chains, regulation, and consumer markets.

## 6 Industrial, regulatory, and sustainability constraints on cocoa butter substitutes

The commercial implementation of CBS is governed by a combination of functional performance requirements, regulatory classification, and increasingly stringent sustainability expectations. However, market size alone does not determine feasibility; CBS must integrate into tightly controlled chocolate manufacturing systems without compromising tempering behaviour, polymorphic stability, or sensory quality.

### 6.1 Functional and regulatory boundaries

CBS are rarely deployed as direct molecular replacements for cocoa butter in premium chocolate. Instead, they are typically incorporated through controlled blending strategies designed to balance cost, functionality, and compliance (Mokbul & Siow, 2022). Even minor deviations in melting range, SFC profile, or crystallization kinetics can induce fat bloom or textural defects, limiting suitability for high-value applications (Afoakwa, 2016; Yang, Lee, et al., 2024). Regulatory frameworks further constrain application domains. In the European Union, Directive 2000/36/EC permits the inclusion of up to 5% specified vegetable fats in chocolate, provided clear labelling is applied (Weber, 2026). In contrast, United States standards of identity restrict the use of the term “chocolate” for products containing alternative fats, effectively segmenting CBS usage towards compound coatings and confectionery fillings (Mokbul & Siow, 2022). These regulatory asymmetries reinforce functional segmentation within the market, where premium chocolate categories remain anchored to authentic cocoa butter (Quelal-Vásconez et al., 2020).

### 6.2 Sustainability and supply-chain pressures

Cocoa production faces escalating environmental and socio-economic pressures, including climate vulnerability, deforestation risk, and farmer livelihood instability (Fountain & Hütz-Adams, 2025; Konar et al., 2026). Emerging governance frameworks such as the EU Deforestation Regulation and associated due diligence legislation impose heightened traceability and land-use verification requirements across agricultural commodities (European Commission, 2023; Weber, 2026). These expectations apply equally to CBS feedstocks. Palm-derived fractions, although industrially established, remain under scrutiny for land-use change and biodiversity impacts (Aumpai et al., 2022). Accordingly, sustainability positioning of CBS increasingly requires rigorous life-cycle assessment (LCA) validation and auditable supply-chain documentation (García-Herrero et al., 2019; Konar et al., 2026). For next-generation biotechnological or microbial CBS, environmental advantage cannot be assumed but must be quantitatively demonstrated relative to conventional cocoa and palm supply chains. Overall, industrial adoption of CBS depends on achieving a triad of criteria: functional equivalence under chocolate processing conditions, regulatory compatibility, and defensible sustainability metrics. These constraints directly shape the technological priorities discussed in subsequent sections.

## 7 Future directions: precision engineering and systems-level integration

The future of CBS development is shifting from approximate replacement towards precision lipid engineering. Across enzymatic modification, microbial synthesis, and plant biotechnology, the central challenge is no longer simply increasing oleic, stearic, or palmitic acid content, but reconstructing CB-like S–U–S TAG architecture with predictable crystallization behaviour under industrial processing conditions. In plant systems, future progress will require coordinated, tissue-specific control of fatty acid biosynthesis, acyl-CoA partitioning, and TAG assembly. Emerging synthetic biology tools, including multiplex CRISPR-based transcriptional regulation and synthetic promoter systems, may enable more precise modulation of key enzymes such as KASII, SAD, FAD2, GPAT, LPAT, and DGAT while reducing pleiotropic effects on membrane lipid homeostasis (Nayak et al., 2025). Microbial platforms are undergoing parallel refinement through dynamic regulatory circuits, feedback-responsive promoters, and systems-level metabolic modeling, which aim to balance precursor supply, growth, lipid accumulation, and TAG stereochemistry in engineered yeasts (Konzock et al., 2022; Wang et al., 2020). These approaches highlight that stereochemical fidelity, rather than altered fatty acid composition alone, will be the defining requirement for next-generation CBS.

A diversified production ecosystem is likely to emerge rather than a single universal solution. Enzymatic interesterification of sustainably sourced plant fractions remains the most commercially mature route for large-scale compound applications (Norazlina et al., 2021). Microbial fermentation offers a programmable platform for compositionally defined, high-value lipids but must still overcome constraints in titre, downstream recovery, food-grade processing, and regulatory approval (Konzock et al., 2022; Wei et al., 2018). Plant biotechnology represents the longer-term ambition of producing structurally precise TAGs directly in scalable oilseed crops. Ultimately, successful CBS development will depend on integrating biochemical precision, processing compatibility, regulatory acceptability, and verified sustainability. Only systems that reproducibly control TAG stereochemistry, crystallization pathways, and environmental performance are likely to progress from complementary ingredients to structurally equivalent lipid solutions for the global chocolate supply chain.

## CRediT authorship contribution statement

**Yuxin Zou:** Writing – review & editing, Writing – original draft, Methodology, Investigation. **Yan Lu:** Resources, Supervision, Visualization, Writing – review & editing. **Changquan Zhang:** Writing – review & editing, Supervision, Resources. **Baolong Zhang:** Writing – review & editing, Supervision, Resources, Project administration, Funding acquisition. **Qing Liu:** Writing – review & editing, Writing – original draft, Supervision, Formal analysis, Conceptualization.

## Consent to publish

All authors have reviewed the manuscript and consent to its publication.

## Ethical approval

This article does not contain any studies with human participants or animals performed by any of the authors. Ethical approval was not required for this review article.

## Funding

This work was supported by Zhongshan Biological Breeding Laboratory under Grant No. ZSBBL-KY2023, the 10.13039/501100012246Priority Academic Program Development of Jiangsu Higher Education Institutions (PAPD), and Jiangsu Qing Lan Project.

## Declaration of competing interest

The authors declare that they have no known competing financial interests or personal relationships that could have appeared to influence the work reported in this paper.

## Data Availability

Data sharing is not applicable to this article as no new data were created or analyzed in this study.
